# Single-Cell Lipidomics: An Automated and Accessible Microfluidic Workflow Validated by Capillary Sampling

**DOI:** 10.1021/acs.analchem.4c03435

**Published:** 2024-10-26

**Authors:** Anastasia Kontiza, Johanna von Gerichten, Kyle D. G. Saunders, Matt Spick, Anthony D. Whetton, Carla F. Newman, Melanie J. Bailey

**Affiliations:** † School of Chemistry and Chemical Engineering, Faculty of Engineering and Physical Sciences, University of Surrey, Guildford GU2 7XH, United Kingdom; ‡ School of Health Sciences, Faculty of Health and Medical Sciences, University of Surrey, Guildford GU2 7XH, United Kingdom; § vHive, School of Veterinary Medicine, School of Biosciences and Medicine, University of Surrey, Guildford GU2 7XH, United Kingdom; ∥ Cellular Imaging and Dynamics, GlaxoSmithKline, Stevenage SG1 2NY, United Kingdom

## Abstract

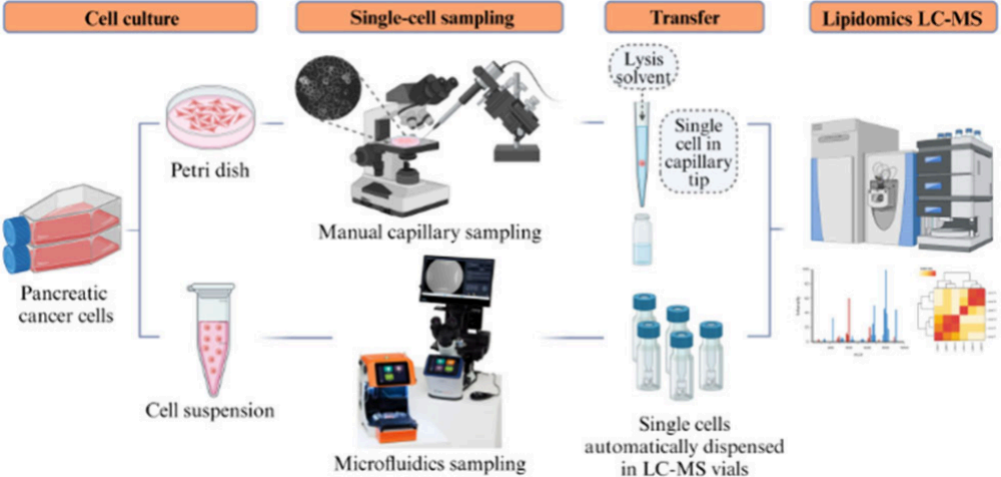

We report the first demonstration of a microfluidics-based approach to measure lipids in single living cells using widely available liquid chromatography mass spectrometry (LC-MS) instrumentation. The method enables the rapid sorting of live cells into liquid chambers formed on standard Petri dishes and their subsequent dispensing into vials for analysis using LC-MS. This approach facilitates automated sampling, data acquisition, and analysis and carries the additional advantage of chromatographic separation, aimed at reducing matrix effects present in shotgun lipidomics approaches. We demonstrate that our method detects comparable numbers of features at around 200 lipids in populations of single cells versus established live single-cell capillary sampling methods and with greater throughput, albeit with the loss of spatial resolution. We also show the importance of optimization steps in addressing challenges from lipid contamination, especially in blanks, and demonstrate a 75% increase in the number of lipids identified. This work opens up a novel, accessible, and high-throughput way to obtain single-cell lipid profiles and also serves as an important validation of single-cell lipidomics through the use of different sampling methods.

## Introduction

Lipids play crucial roles in various biological processes, including cell membrane structure, signaling pathways, energy storage, and metabolism.^1,2^ As such, lipidomics, the comprehensive study of lipid molecules within a biological system, has emerged as a vital field in modern biology and medicine. Traditional bulk lipid analysis methods, while informative, often fail to capture the heterogeneity present within a cell population, leading to insufficient understanding of lipid dynamics at the cellular level.^3^ Additionally, unlike transcriptomics studies which have benefited from amplification methods,^4^ our knowledge of the precise physiological roles of small molecules at the cellular level, such as lipids, remains limited. Therefore, considering the genetic and phenotypic variability exhibited by cell populations, probing the lipidome at the single-cell level in a sensitive and reliable manner is crucial for characterizing important cellular states, such as growth, differentiation, and aging. The information provided by single-cell lipidomics research holds wide potential for impact, including drug discovery and the treatment of infectious diseases, allergy, and cancer, which exhibit highly heterogeneous conditions.

Mass spectrometry is the preferred method for detecting lipids in biological tissues as well as in single cells due to the combination of sensitivity and specificity at low concentrations and the ability to perform comprehensive analysis of a wide range of lipid classes. Recent advancements in mass spectrometry have enabled single-cell lipidomics, aiming to unravel the heterogeneity and complexity of cellular lipid metabolism with unprecedented sensitivity. Single-cell lipidomics approaches include mass spectrometry imaging with single-cell spatial resolution, such as matrix-assisted laser desorption/ionization (MALDI), secondary-ion mass spectrometry (SIMS), and desorption electrospray ionization (DESI), which can rapidly sample single cells in tissue, or fixed cells and obtain lipid profiles.^5−14^ A limitation of these approaches is that the lack of chromatographic separation can lead to matrix effects that cause ionization suppression and difficulty identifying isomers. Additionally, cells are not sampled in their native state due to the need for cryopreservation, freeze dehydration, or fixation prior to analysis.

In contrast to mass spectrometry imaging, single-cell sampling (SCS) techniques can be used to sample living cells and include capillary sampling and microfluidics-based cell sorting. Capillary sampling uses microscopy for the aspiration of single living cells into glass capillaries under negative pressure. It is a powerful technique which allows for the preservation of spatial information and has previously been demonstrated in conjunction with liquid chromatography–mass spectrometry (LC-MS) for untargeted lipidomics and metabolomics.^15−19^ Microfluidics-based techniques can offer a low-cost alternative for live single-cell analysis when the speed of sampling is preferred over spatial information. They allow precise manipulation and control of small volumes of fluids and the isolation of individual living cells. Microfluidics has primarily been demonstrated in conjunction with single-cell proteomics and genomics,.^20−22^ Single-cell lipidomics studies employing microfluidics are few and do not utilize LC separation prior to detection.^23,24^ To our knowledge, the integration of microfluidic single-cell sampling with liquid chromatography has yet to be reported.

Despite the potential of microfluidic sampling techniques, several challenges remain for their integration with mass spectrometry lipidomics. These challenges include the efficient isolation of single live cells, the extraction and separation of lipids from minute sample volumes, and the compatibility with liquid chromatography–mass spectrometry. Addressing these challenges requires the optimization of SCS techniques to ensure robust and reliable single-cell lipidomics analysis.

In this study, we aim to integrate and optimize a microfluidics-based technique by Soitu et al.^25−28^ into a single live-cell mass spectrometry lipidomics workflow. We also benchmark the optimized microfluidics method against a capillary sampling method, through comparison of lipid coverage. Our overarching goal is to provide researchers with a robust and versatile workflow for investigating cellular lipid metabolism at the single-cell level, thereby advancing our understanding of lipid-related diseases and facilitating the development of targeted therapeutic interventions.

## Experimental Section

### Cell Culture

Human pancreatic adenocarcinoma cells, PANC-1 (Merck, U.K.), were cultured in Corning T25 culture flasks (Merck, U.K.) with Dulbecco’s modified eagle medium (DMEM) with glucose (Sigma-Aldrich, U.K., Cat. No. 21969035), 10% (*v*/*v*) fetal bovine serum (FBS) (Fisher Scientific, U.K., Cat. No. 11550356), 1% penicillin/streptomycin (Fisher Scientific, U.K., Cat. No. 15140122), and 2 mM l-glutamine (Sigma-Aldrich, U.K., Cat. No. 25030024). The cells were incubated at 37 °C in 21% *v*/*v* O_2_ and 5% *v*/*v* CO_2_. The cell culture medium was replaced every other day and cells were split every 3 days when the culture reached approximately 80% confluency. In preparation for single-cell sampling, approximately 250000 cells were seeded into a sterile T25 flask or BioLite cell-culture treated dishes (Thermo Fisher Scientific, U.K., Cat. No. 130181) for microfluidics and capillary sampling, respectively. Additionally, for capillary sampling, an equivalent volume of cell culture medium without cells was aliquoted into separate sterile dishes and treated as blanks.

On the day of single-cell sampling, cells were washed three times with warm Dulbecco’s phosphate-buffered saline (PBS) to remove any residual culture medium. Subsequently, the cells were maintained in either warm FBS-containing or FBS-free media throughout the cell sampling process. For microfluidics sampling, a single cell suspension was created using Gibco trypsin-EDTA (Fisher Scientific, U.K., Cat. No. 10779413), incubated for 5 min at 37 °C in 21% O_2_ and 5% CO_2_, followed by centrifuging (1500 rpm, RT) and resuspending the cell pellet in warm FBS-containing or FBS-free media, with frequent mixing to avoid cell aggregation.

### Microfluidics-Based Automated Single-Cell Picking

The single-cell (sc) picking platform from iotaSciences is a modular system. Here, we made use of the following components: a fluid handler (isoPick), a microscope (isoHub), and an imaging system. To dispense single cells from a suspension into individual chambers, a 6 cm Petri dish was first coated with 2 mL of an albumin-based buffer (iotaSciences, U.K.). This was then removed, leaving a thin film of the buffer wetting the base of the dish. The film of buffer was overlaid with 2 mL of immiscible fluorocarbon HF^BIO^ (iotaSciences, U.K.). The dish was then slotted in the dish holder on the isoPick, whereby the automatic formation of 256 optically clear liquid chambers (GRIDs, 3.24 mm^2^ area) was completed, using a fluid microjet to create fluid walls that physically isolated each chamber. Creating a GRID took approximately 3 min.

Cells from a cell media suspension (∼15000 cells per mL) were dispensed into the GRID, and the presence of a single cell was confirmed using the isoHub with a 10× objective to manually visualize every GRID chamber. Chambers containing one single cell were selected, and location coordinates were automatically saved in both modules of the picking platform. The isoPick then automatically aspirated the selected chambers containing a single cell and dispensed them into Qsert LC-MS vials (Waters, U.K., Cat. No. 186001126DV).

The total volume of cell, HF^BIO^, and PBS was ∼1.5 μL, which was made up to 15 μL by manual addition of the cell lysis solvent, which was a mixture of EquiSPLASH (16 ng/mL, Avanti Polar Lipids, Cat. No. 330731) diluted in the initial mobile phase composition (70:30 A/B), and supplemented with 0.01% butylated hydroxytoluene (BHT, Fisher Scientific, U.K., Cat. No. 11482888) to prevent lipid oxidation. The vials were then capped and stored at −80 °C until the day of analysis.

### Optimization of the iotaSciences Single-Cell Picking Platform

Ammonium formate solutions were prepared using LC-MS grade water (Chromasolv Honeywell, Fisher Scientific) and ammonium hydroxide solution (>99%, ROMIL-SpA, U.K., Cat. No. HB059T), which were sterile filtered. Each solution was fixed to pH 7.4. PANC-1 cells were cultured in BioLite 96-well plates (Thermo Fisher Scientific, U.S.A., Cat. No. 130188), with media prepared as described above. When the culture reached 80% confluency, the media was removed, and wells were washed 3× with PBS. Each concentration of ammonium formate solution (pH 7.4) was tested in triplicate. The cells were left in the solutions for 10 min (maximum time of contact during microfluidics sampling), and a standard trypan blue (Thermo Fisher Scientific, U.S.A., Cat. No. 15250061) cell viability assay was performed.^29^

All optimization experiments took place on the same day with cells of the same passage number and were analyzed on the same LC-MS run. A typical sampling experiment with 45 cells sampled in vials took 90 min.

### Manual Live-Cell Capillary Sampling

Capillary sampling was conducted under ambient conditions, using the method described by Lewis et al.^17^ Cells of comparable diameter were sampled into 10 μm capillary tips (Yokogawa, Japan) in FBS-free media, and thus, each cell capture carried a small volume of media. Next, 5 μL of the cell lysis solvent was added to the back of each tip using a 5 μL syringe (Hamilton, U.K., Part No. 87919). The filled tips were immediately placed on dry ice and then stored at −80 °C overnight. Blank samples were provided by drawing media into capillary tips. Dispensing the samples from the capillaries into Qsert LC-MS vials was performed with a gas syringe fitted with a Luer-lock adapter (65 μL/min flow rate), as described previously.^15^ An additional aliquot of the lysis solvent (10 μL) was then added to each vial to make a total volume of 15 μL per sample. The vials were then capped and stored at −80 °C until the day of analysis. A typical experiment with 40 single cells sampled in tips took 5 h.

### Lipidomics LC-MS Analysis

Lipids were detected using an Ultimate 3000 UHPLC (Thermo Fisher Scientific, U.S.A.) system coupled to a Q-Exactive Plus Orbitrap (Thermo Fisher Scientific, U.S.A.) mass spectrometer; Full-scan MS method described previously.^15^ In brief, the ionization source (HESI) probe was set to 320 °C with a spray voltage of 4 kV, automatic gain control (AGC) target of 1 × 10^6^, mass range of 200–1400 *m*/*z*, and a resolution setting of 140000. Data were acquired in positive ionization mode.

The total 15 μL volume in samples was injected into a C30 column (Accucore, 2.6 μm, 2.1 × 150 mm, Thermo Fisher Scientific) at 40 °C with a flow rate of 0.35 mL/min. The solvent systems (LC-MS grade, Chromasolv Honeywell, Fisher Scientific) were **A** 60:40 (*v*/*v*) acetonitrile/water and **B** 85:10:5 (*v*/*v*) isopropanol/water/acetonitrile, both containing 0.1% (*v*/*v*) formic acid (LC-MS grade, Fisher Chemical Optima, Fisher Scientific) and 10 mM ammonium formate (99%, Acros Organics). The LC gradient information is provided in the Supporting Information (Table S1).

### Data Analysis

Lipostar 2 (Molecular Discovery, Italy) was used to process the data files (.raw). A 3× signal/noise ratio filter based on mass spectrum signal intensity was used before lipid identification. Gap filling was disabled due to the heterogeneity of single cells.

Only peaks assigned to lipid classes were represented in the internal standard (EquiSPLASH: PC, LPC, PE, SM, Cer, PG, TAG, DAG, PS, LPE, PI, MG, and Chol Ester) and their ether forms were selected for further analysis. Blank subtraction, 3× signal/noise ratio filtering based on peak area, normalization to the internal standard, and handling of data was conducted using Freestyle (Thermo Fisher Scientific, U.S.A.) and Excel (Microsoft, U.S.A.) software. Data were log transformed and autoscaled, with zero values replaced with half the minimum value of each sample, before multivariate statistical analysis using MetaboAnalyst 5.0.^30^ GraphPad Prism version 8.4.3 (Windows, GraphPad Software, U.S.A.) was used for creation of plots and *t* test and f-test calculations. The code editor VS Code version 1.78.0 (Anaconda Navigator GUI for Windows, version 2.3.2) was used to produce heatmaps using the following libraries: pandas, numpy, seaborn, and matplotlib. Principal component analysis (PCA) was performed using RStudio (R version 4.2.1) using the libraries factoextra and readr.

Where “average” number of features are reported, this refers to the mean number of lipid features detected (as per the criteria above) per single cell or blank sample. In contrast, the “total” number of features refers to the sum of the lipid features detected in each sample of a group.

## Results and Discussion

**Figure 1:**
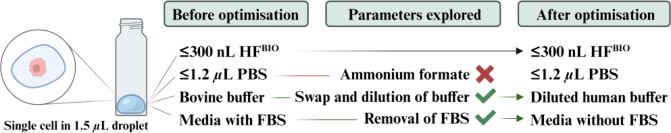
Schematic showing the composition of the liquid droplets sampled with the single-cell microfluidics sampling method, before and after optimization, as well as the parameters explored. Created using BioRender.com.

This study reports the successful integration and optimization of a microfluidics-based technique for isolating single live cells to a lipidomics LC-MS method. The parameters explored are summarized in Figure 1, and a schematic showing the instrumentation is available in Supporting Information, Figure S1. A direct comparison of the microfluidics method to manual single-cell capillary sampling was performed to compare the lipid coverage.

### Manufacturers’ Protocol for Microfluidics Sampling Yields High Lipid Contamination in Blank Samples

To couple the scPicking Platform (iotaSciences, UK) to single-cell lipidomics, we first isolated single live PANC-1 cells in LC-MS vials using the protocol recommended by the manufacturers (summarized in Figure 1, left). Specifically, the cell suspension was prepared in media containing fetal bovine serum (FBS), GRIDs were prepared using a bovine-based albumin buffer (iotaSciences, U.K.) and dispensing of the cells was facilitated with the use of PBS. The volume of the droplet deposited into the Qsert vials was estimated to be 1.5 μL, of which ≤1.2 μL was PBS and ≤300 nL was HF^BIO^ (iotaSciences, U.K.), with trace amounts of FBS-containing media, and buffer.

**Figure 2:**
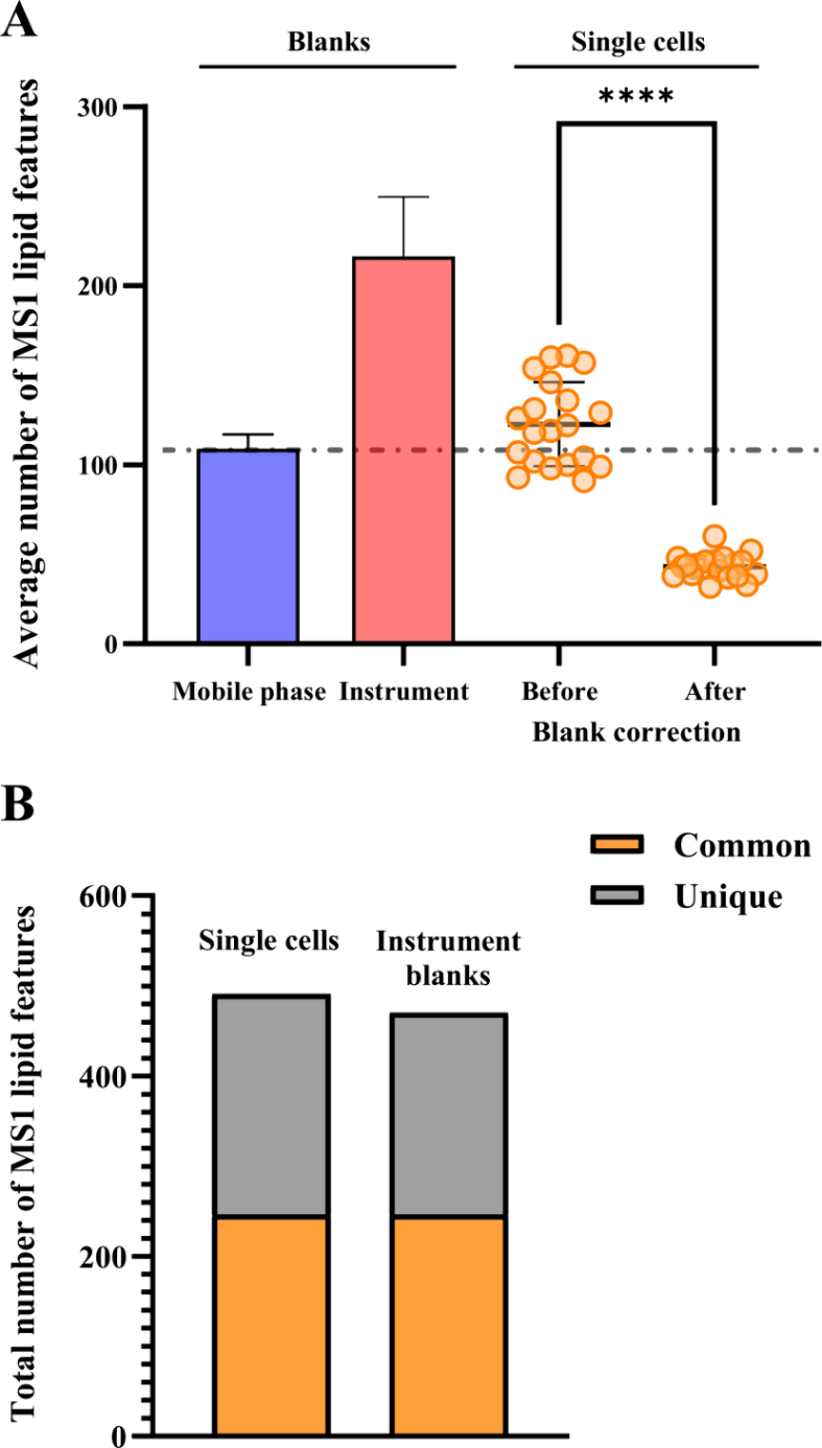
Unoptimized method. (A) Average number of lipid features detected in mobile phase blanks (*n* = 3), instrument blanks (*n* = 5) and PANC-1 cells (*n* = 20) isolated with the microfluidics method, before and after blank correction. An unpaired *t* test performed for the single cell sample data, before and after blank correction, produced a p value of <0.0001. Error bars = 1 SD. (B) Total number of common and unique lipid features detected in single PANC-1 cells (*n* = 20), isolated with the microfluidics method, compared to the instrument blanks (*n* = 5).

Figure 2A)shows the numbers of lipid features detected in the initial experiments, prior to optimization. A higher number of lipid features was detected in the blank samples obtained from the microfluidics instrument (“instrument blanks”) than in the mobile phase blanks (70:30 mobile phase A/B). This resulted in a significant drop (*p* < 0.0001) in the average number of lipid features detected in single cells after correction to the instrument blanks. A relatively low average number of 43 lipid features per single cell was detected, lower than achieved by other methodologies for the same cell type, of around 200 lipid features.^15,16^ The same effect can be seen in Figure 2B when comparing the total numbers of common and unique lipid features seen in the instrument blanks and the single cell samples. Around 50% of the lipid features found in cells were also detected in the instrument blanks, leading to the removal of those features after blank correction. Our data shows how the purity of the blank sample plays an important role in the detection of lipids from single cells. Therefore, optimization was carried out to reduce lipid contamination in the microfluidics blanks.

### Elimination of Lipid Contamination and Ionization Suppression Sources Results in Improved Detection of Lipids in Single Cells

The objective of the optimization was to reduce lipid signals in the instrument blanks, thereby improving the sensitivity to lipids in single cells. The parameters selected for optimization included: (I) replacing the cell-culture media with FBS-free media; (II) replacing the bovine-derived albumin buffer used to coat the Petri dish before formation of GRIDs with a purer, human-derived albumin buffer and diluting 10-fold, with the aim of reducing the lipid content of the blanks; and (III) replacing the PBS with ammonium formate (150 mM, pH 7.4) to reduce possible ion suppression from PBS.

**Figure 3:**
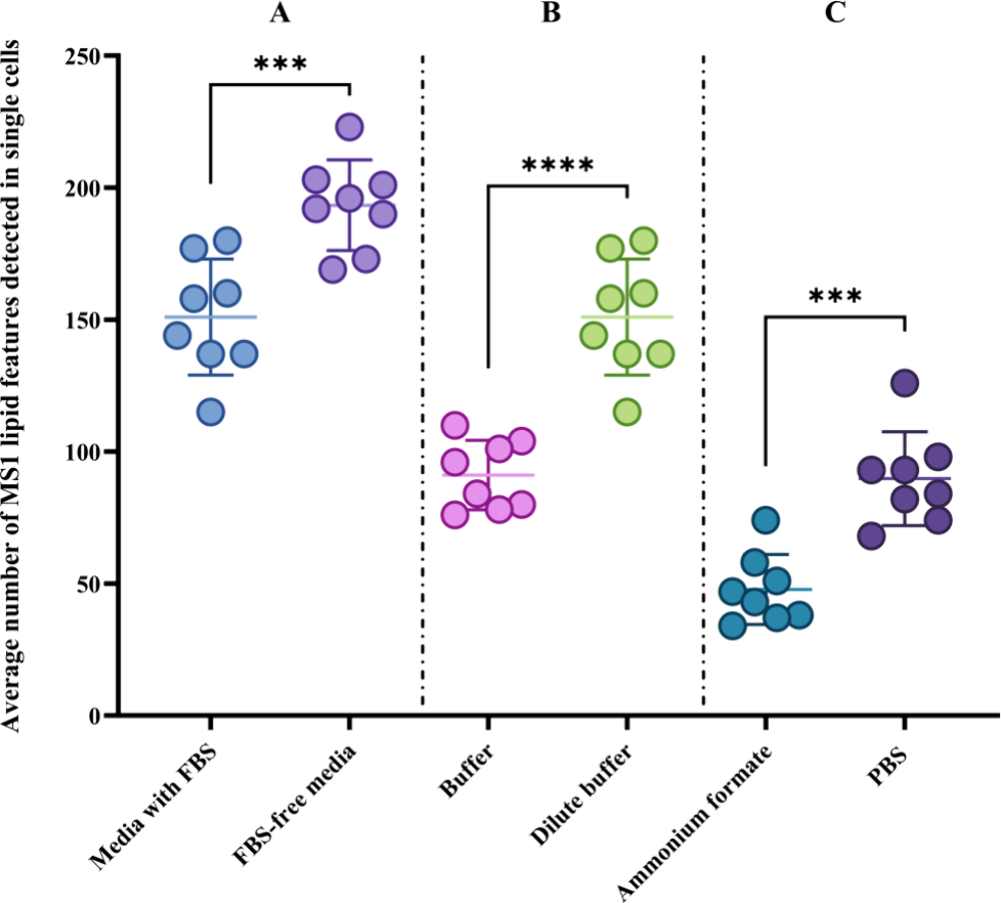
Difference in the average number of lipid features detected in PANC-1 cells isolated with the microfluidics method. *N* = 8 for all groups. Data is blank corrected using instrument blanks from the microfluidics method. Error bars = 1 SD. (A) Using fetal bovine serum-containing media or fetal-bovine serum-free media, *p* = 0.0007. (B) Human-derived albumin buffer or 1:10 diluted human-derived albumin buffer, *p* < 0.0001. (C) Using ammonium formate or phosphate-buffered saline, *p* = 0.0001.

Figure 3A shows the effect of removing FBS from the media in which the PANC-1 cells were suspended before dispensing into GRIDs. There is a significant increase in the number of lipid features detected in single cells after blank correction when removing FBS from the media. This is due to the significantly higher number of lipids (*p* = 0.01) detected in the instrument blanks containing media with FBS, than in blanks containing no FBS (Supporting Information, Figure S2). In Figure 3B, the effect of a 10× dilution of the human-derived albumin buffer is shown. The results show a clear increase in detected lipids when diluting the buffer used to coat the Petri dishes before formation of GRIDs and single-cell dispensing (*p* < 0.0001). The 10-fold dilution resulted in a significant decrease (*p* = 0.0006) of lipid features detected in the instrument blanks (Supporting Information, Figure S3). Further dilution of the buffer resulted in inconsistent formation of the liquid chambers, and thus failed isolation of cells.

To assess whether the PBS used during microfluidics sampling of single cells could be replaced with an alternative carrier solvent to better aid ionization, a series of ammonium formate concentrations between 10 and 170 mM were tested (Supporting Information, Figure S4). Using a standard cell viability assay based on Strober et al.^29^ we determined that a concentration of 150 mM of ammonium formate fixed to physiological conditions (pH 7.4) was the best condition for preservation of cell viability (97%, after 10 min of exposure). Figure 3C shows the number of lipid features detected in PANC-1 cells obtained by replacing phosphate-buffered saline (PBS) with ammonium formate. Surprisingly, PBS remained favorable as a greater number of lipids were detected (*p* = 0.0001). It was observed that during the single-cell isolation process there was a partial blockage of the dispensing needle when ammonium formate was used in place of PBS. This led to the gradual reduction in the volume of droplets dispensed in vials and a lower recovery of lipids.

### Optimized Microfluidics Parameters Increase the Number of Lipids Detected in Single Cells

**Figure 4:**
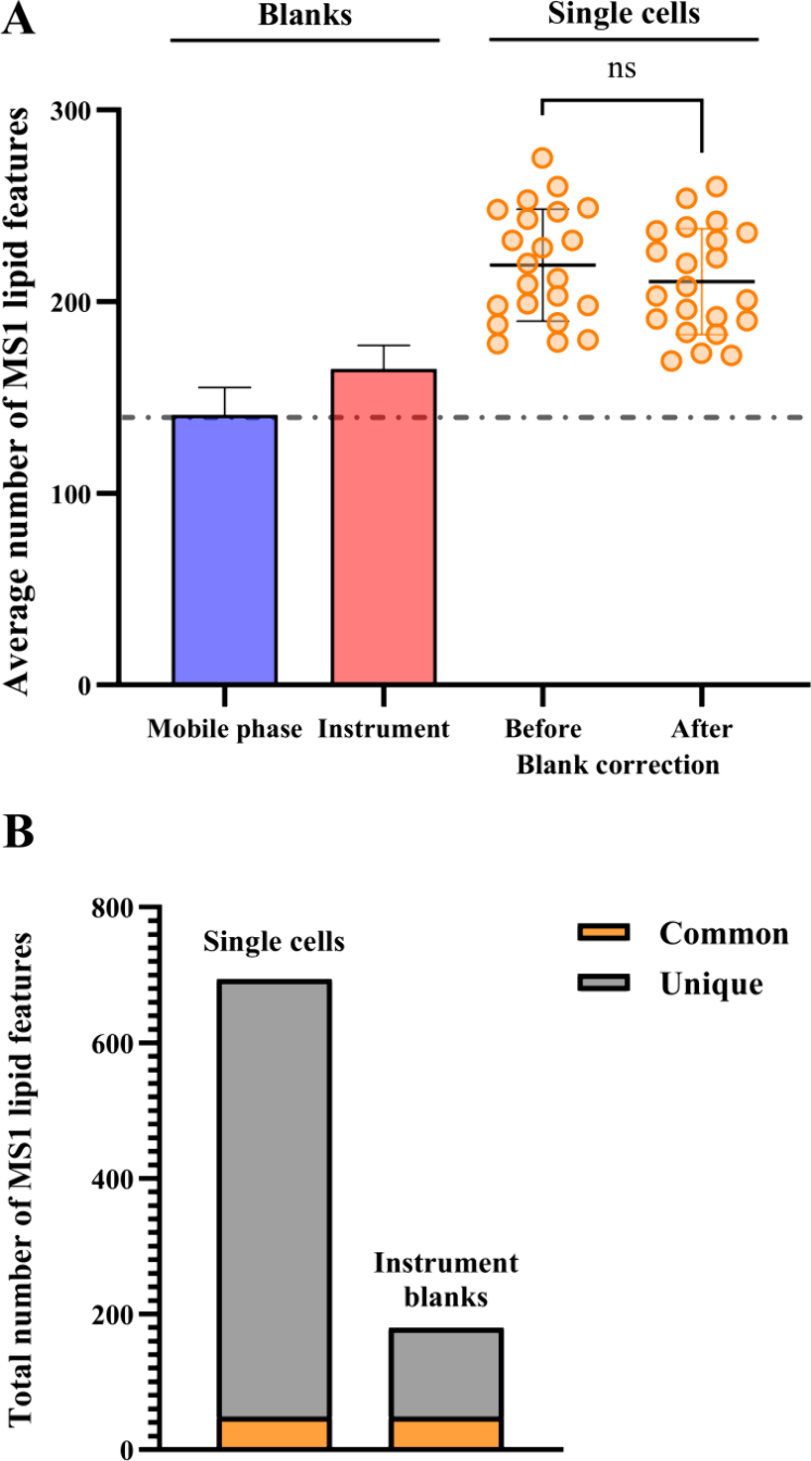
Optimized method. (A) Average number of lipid features detected in mobile phase blanks (*n* = 3), instrument blanks (*n* = 5), and PANC-1 cells (*n* = 20) isolated with the microfluidics method, before and after blank correction. Error bars = 1 SD. (B) Total number of common and unique lipid features detected in single PANC-1 cells (*n* = 20), isolated with the microfluidics method, compared to the instrument blanks (*n* = 5) after optimization.

Figure 4 shows single-cell lipidomics data from cells sampled using the scPicking Platform with the optimized parameters for single-cell lipidomics (FBS-free media, dilute human-based albumin buffer, and dispensing of cells in PBS). The composition of the droplets is summarized in Figure 1.

The results confirm the successful reduction in lipids detected in the instrument blanks after optimization, as shown in Figure 4A. Prior to optimization, 50% more lipid features were detected in the instrument blanks than the mobile phase blanks reducing to 15% after optimization. Thus, when correction to the instrument blanks was applied, the average number of lipid features per single cell detected (210) did not differ significantly from the average number before correction (219). Figure 4B depicts the total number of common and unique lipid features detected in the instrument blanks and the single cell samples. Comparing Figure 4B and Figure 2B, it is clear that the optimization steps undertaken resulted in a significant reduction in lipid features common to the instrument blanks and single-cell samples.

### Comparison of Optimized Microfluidics Method to Established Capillary Sampling of Live Single Cells Shows Same Performance

Single live PANC-1 cells were sampled using the optimized microfluidics sampling method and a capillary sampling method previously reported with LC-MS for detection of lipids in single cells.^15,17^

**Figure 5:**
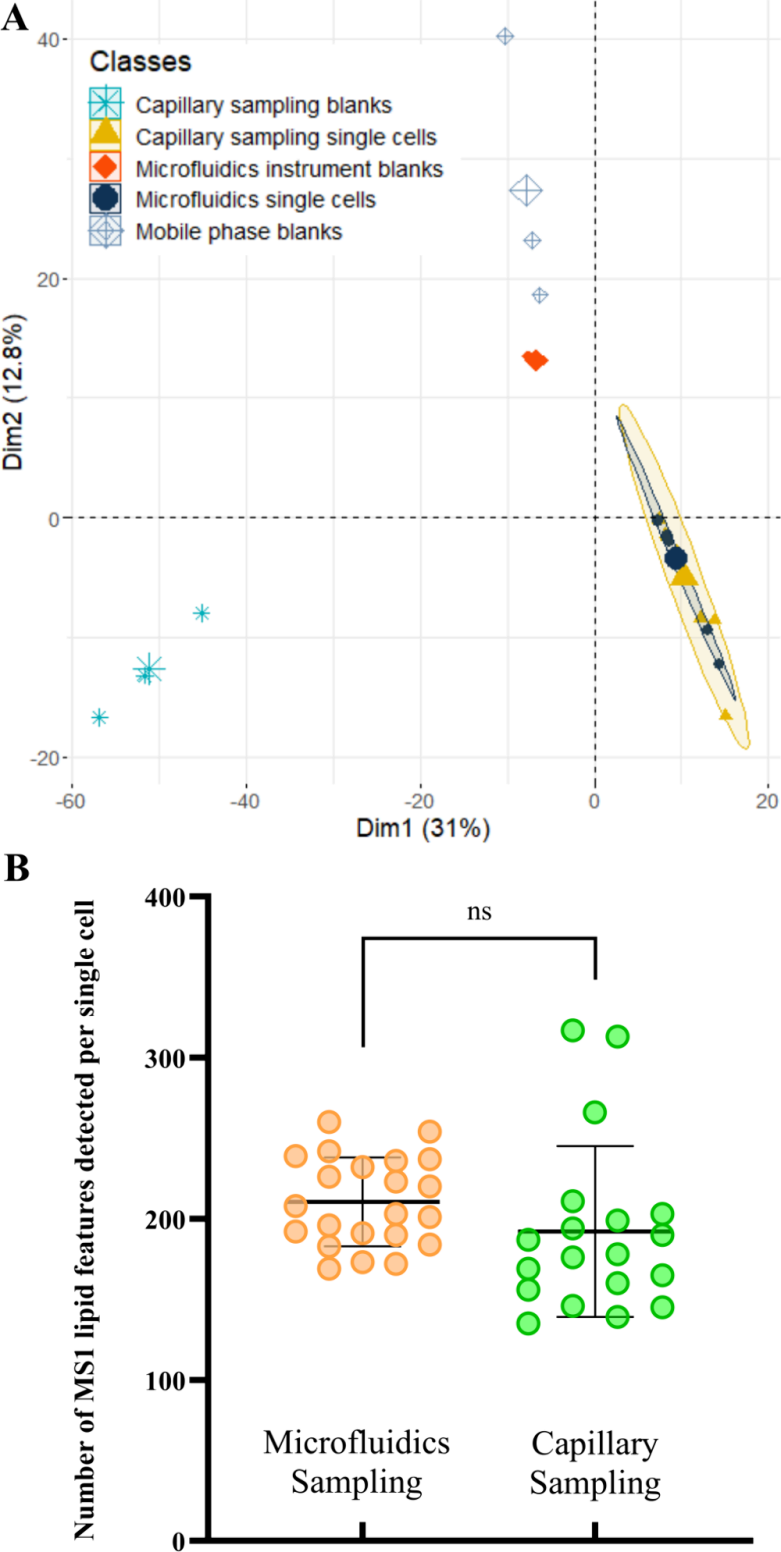
Comparison of optimized microfluidics single live-cell sampling to capillary sampling. (A) PCA of the lipid profiles of capillary sampling blanks (*n* = 3), microfluidics instrument blanks (*n* = 3), mobile phase blanks (*n* = 3), microfluidics sampling single cells (*n* = 10), and capillary sampling single cell samples (*n* = 10). (B) Average number of lipid features detected per single cell using microfluidics sampling (*n* = 20) and capillary sampling (*n* = 20) to isolate single live PANC-1 cells. An unpaired *t* test performed for the single cell sample data produced a *p* value of 0.1624. Error bars = 1 SD.

The PCA plot is shown in Figure 5A shows a clear separation between the various blanks and single-cell samples, with the microfluidics and capillary-sampled cells clustering together. There is also separation of the blank samples produced from capillary sampling and microfluidics. This can be explained by different lipid contaminants being present in the capillary tips compared to those in the microfluidics system. The PCA also shows the microfluidic instrument blanks clustering more closely to the mobile phase blanks than the capillary sampling blanks. This could be due to contaminants present in the tips used but also the amount of culture media sampled. This observation highlights the similarity of single-cell lipid profiles obtained with these two complementary sampling methods.

As seen in Figure 5B, between microfluidics and capillary sampling, the average number of lipids (211 ± 27 and 192 ± 52, respectively) detected per single cell is not statistically different. This highlights the methods’ ability to obtain comparable lipid signatures. However, an F-test showed that the variation in the number of lipids recovered per single cell was greater for capillary sampling (*p* = 0.0051). Our analysis of the internal standards also showed that the variability of the microfluidics sampling method was statistically lower (*p* = 0.0015) than that of capillary sampling (see Table S2). One possible explanation for these two observations is that capillary-sampled cells are manually transferred into vials, resulting in higher variation compared to automated transfer of single cells using the microfluidics method. This highlights an area of future optimization for capillary sampling.

**Figure 6:**
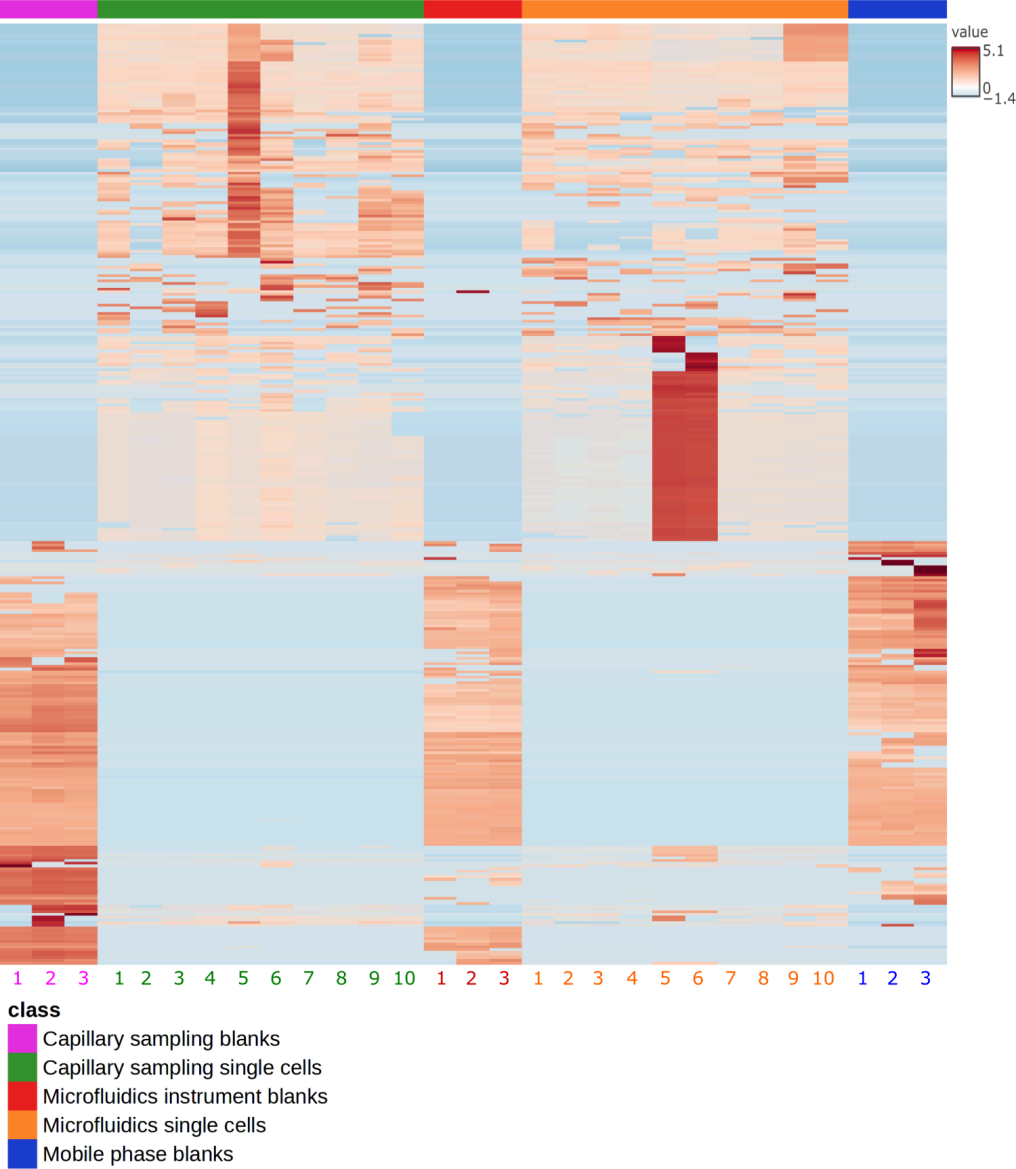
Clustered heatmap of lipidomics single-cell samples and various blanks (log transformed and auto scaled). Only lipids that are present in at least 50% of samples per group are shown. *N* = 10 for capillary sampling single cells and microfluidics single cells. *N* = 3 for mobile phase, microfluidics, and capillary sampling blanks.

Figure 6 is a clustered heatmap, showing only the lipid features that are present in at least 50% of the sample groups (specifically, capillary-sampling single cells, microfluidics-sampling single cells, mobile phase, and instrument blank samples). A version without a 50% frequency filter can be seen in Supporting Information, Figure S5, and a by-class distribution of all lipid features detected in blanks and single-cell samples can be found in Figures S6 and S7.

The heatmap clearly shows that the vast majority of lipid features found in the various blank samples are different from the ones in the single cell samples. This highlights the ability of the two single-cell sampling methods to perform single-cell lipidomics. Interestingly, the single-cell sampling methods showed similar lipid signatures. This can be expected due to the sampling of the same cell line from the same passage number, on the same day. The fact that the optimized microfluidics sampling method reveals similar lipid profiles to the already-established capillary sampling method means that users interested in single-cell lipidomics research can now access an additional workflow which is ∼80% faster, to profile the lipidome at the single-cell level, when spatial information is not required. In contrast, capillary sampling provides spatial information.

To the best of our knowledge, this is the first comparison of single-cell lipid profiles using complementary sampling methods. The heatmap in Figure 6. suggests that both methods can detect cell-to-cell variation. To test this, we decoupled method variability from analyte heterogeneity by performing F-tests on the lipids detected in single cells and their corresponding internal standard lipid classes. This confirmed that the analyte variability was greater than the internal standard variability (*p* < 0.0001). We ascribe this result to single-cell heterogeneity, which underlines the necessity of developing sensitive single-cell analysis methods to probe the unique events taking place in cell populations.

We have chosen to report only lipids belonging to the lipid classes found in the internal standard, which impacts the number of lipid features reported. We also acknowledge that the LC-MS instrumentation used for this study does not necessarily provide the best available structural specificity, but as shown by our data, it is sufficient for showing the differences and similarities between the SCS methods discussed and for detecting single-cell heterogeneity.

The importance of single-cell analysis has been repeatedly highlighted and an increasing number of studies conducted have provided insight in the dynamic profiles of cells, their signaling pathways and disease progression.^31−33^ Future applications of single cell lipidomics are wide-ranging and include; drug discovery, for example probing the heterogeneity in cellular response to drug treatment; gaining insight into infectious diseases by probing the metabolism of infected versus uninfected cells and probing the localized responses of cancer cells to radiation to enhance treatments. Thus, it is crucial to keep improving single-cell sampling methods to enhance the granularity of results obtained. Considering that single cells are minute and every step to gain sensitivity can be beneficial to uncovering more information, we recommend that future work explores the application of this methodology to other cell lines (adherent and suspension), the possibility to reduce the noise from instrument blanks further by washing capillary tips, limiting the dilution of cell samples to reduce ionization suppression, and limiting the effect of blank correction in single-cell signatures. Finally, ensuring the stability and sensitivity of the LC-MS instrumentation requires investigating and optimizing solvent purity, as well as standardization of the appropriate cleaning procedures to attain single cell sensitivity.

## Conclusions

We have successfully optimized a rapid single live-cell microfluidics sampling method and integrated it with lipidomics LC-MS. We also compared the optimized method to previously established capillary sampling, showing reproducible lipidomics results independent of the method. This study has addressed the importance of limiting contamination in blank samples, which, if adopted, allows for comprehensive profiling of single cells. The choice of cell isolation method depends on the biological application. If users require spatial context for the single cells selected, then capillary sampling methods are well suited. However, if high throughput and ease of operation are a priority, as well as the automated transfer of the samples in vials, microfluidics sampling is an excellent alternative.

## Data Availability

The RAW data can be accessed at Zenodo Repository, DOI: 10.5281/zenodo.13710177.

## Supporting Information Available

Table S1: Liquid chromatography gradient. Figure S1: Schematic showing the instrumentation of the scPicking platform. Figure S2: Number of lipid features in blanks for FBS and FBS-free media. Figure S3: Number of lipid features in blanks for buffer and dilute buffer. Figure S4: Ammonium formate concentrations tested. Figure S5: Heatmap of all lipids detected in sampling method comparison experiment. Figure S6: Heatmap of all lipids detected in blank samples sorted by lipid class. Figure S7: Heatmap of all lipids detected in single-cell samples sorted by lipid class. Table S2: Relative standard deviations for internal standard lipid classes (PDF)
Lipid IDs_50% filter (XLSX)
Lipid IDs_no filter (XLSX)
Lipid IDs_raw (XLSX)
