# Supplementary material for: Single-Cell Lipidomics: An Automated and Accessible Microfluidic Workflow Validated by Capillary Sampling
Source: Anal Chem. 2024 Oct 26;96(44):17594–601. doi: 10.1021/acs.analchem.4c03435 (PMC11541894; doi:10.1021/acs.analchem.4c03435)
Supplement: Supplementary file 1 — ac4c03435_si_001.pdf [file ac4c03435_si_001.pdf]

Supporting Information

Single-cell lipidomics: an automated and accessible microfluidic workflow validated by capillary sampling.

Anastasia Kontiza[1], Johanna von Gerichten[1], Kyle D. G. Saunders[1], Matt Spick[2], Anthony D. Whetton[3], Carla F. Newman[4], and Melanie J. Bailey[1*].

1 School of Chemistry and Chemical Engineering, Faculty of Engineering and Physical Sciences, University of Surrey, GU2 7XH Guildford, UK

2 School of Health Sciences, Faculty of Health and Medical Sciences, University of Surrey, GU2 7XH Guildford, UK

3 vHive, School of Veterinary Medicine, School of Biosciences and Medicine, University of Surrey, Guildford, GU2 7XH, UK

4 GlaxoSmithKline, Cellular Imaging and Dynamics – Stevenage, SG1 2NY, UK

*Correspondence:

Corresponding Author: m.bailey@surrey.ac.uk

Table of Contents:

Cover page and table of contents	S1-2
Table S**1**: Liquid chromatography gradient used in LC-MS experiments.	S3
Figure S**1**: Schematic showing the instrumentation of the scPicking Platform	S3
Figure S**2**: Optimisation experiment for media: Average number of lipid features detected in mobile phase blanks.	S4
Figure S**3**: Optimisation experiment for buffer: Average number of lipid features detected in mobile phase blanks.	S4
Figure S**4**: Ammonium formate (pH 7.4) solutions tested with PANC-1 cells for 10 min.	S5
Figure S**5**: Heatmap of single-cell and blank-sample lipidomics data for sampling method comparison.	S6
Figure S**6**: Heatmap of blank-sample lipidomics data, by lipid class.	S7
Figure S**7**: Heatmap of single-cell sample lipidomics data, by lipid class.	S7
Table S**2**: Relative standard deviations for internal standard lipid classes.	S8

*Table S**1**: Liquid chromatography gradient used in LC-MS experiments. Mobile Phase **A** 60:40 (v/v) acetonitrile/water and mobile Phase **B** 85:10:5 (v/v) isopropanol/water/acetonitrile, both containing 0.1 % (v/v) formic acid and 10 mM ammonium formate; linear gradient with a flow of 0.35 mL/min.*

Time (min)	% A	% B
0.0	70	30
5.0	70	30
5.1	57	43
14	30	70
14.1	30	70
21	1	99
24	1	99
24.1	70	30
28	70	30

*Supplementary Figure S**1**. Schematic showing the instrumentation of the scPicking Platform (iotaSciences, UK). (**A**) isoPick™ where all fluid handling, including GRID formation and cell dispensing is achieved (**B**) isoHub™ allowing the visualisation of GRIDs and plated cells (**C**) the LC-MS vial set up where single cells are dispensed into using the isoPick™ and the GRID after sampling of cell droplets have been collected. For the purposes of the schematic, trypan blue was used to create the GRID chambers. Created using Biorender.com*

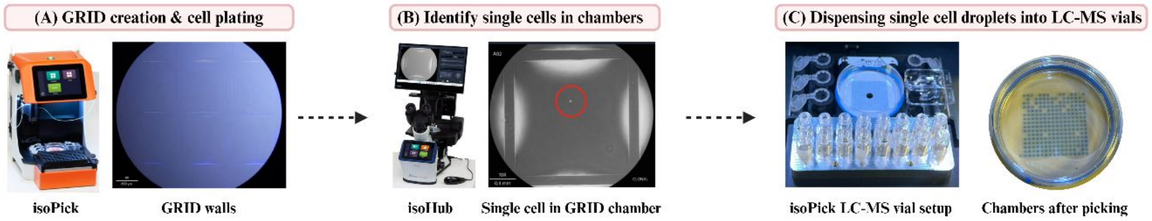

Supplementary Figure S2. Optimisation experiment for media: Average number of lipid features detected in mobile phase blanks (n = 5), instrument blanks containing media with foetal-bovine serum (n = 5), and instrument blanks containing media without foetal-bovine serum (n = 5). The t-test resulted in a p value of 0.0143.

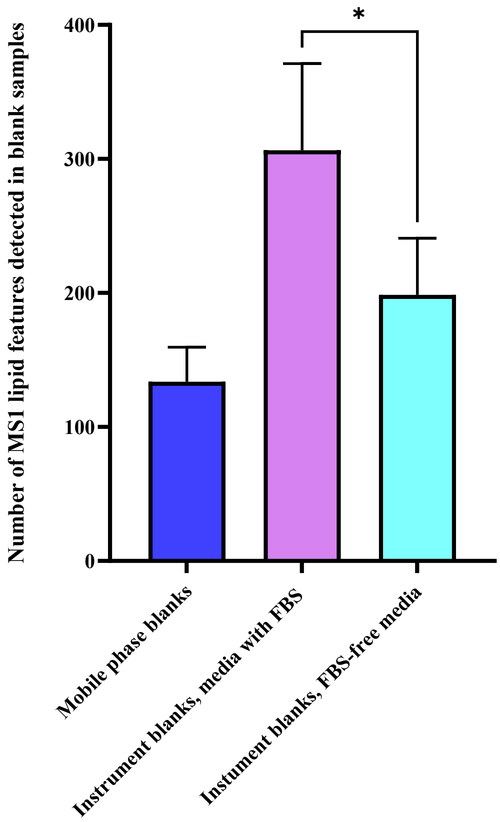

Supplementary Figure S3. Optimisation experiment for buffer: Average number of lipid features detected in mobile phase blanks (n = 5), instrument blanks containing the original concentration of human-based buffer (n = 5), and instrument blanks containing buffer diluted 10 times (n = 5). The t-test resulted in a p value of 0.0006.

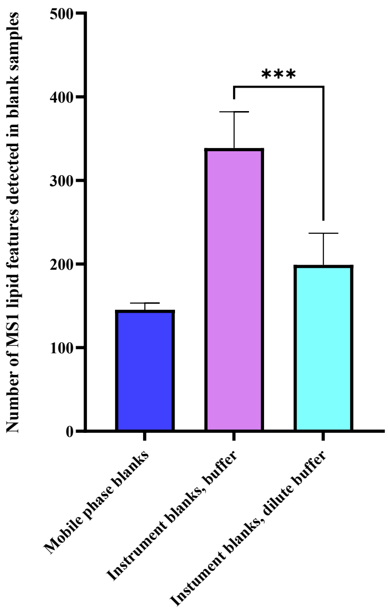

Supplementary Figure S4. Ammonium formate (pH 7.4) solutions tested with PANC-1 cells for 10 min. Showing percentage viability for each concentration. N = 3.

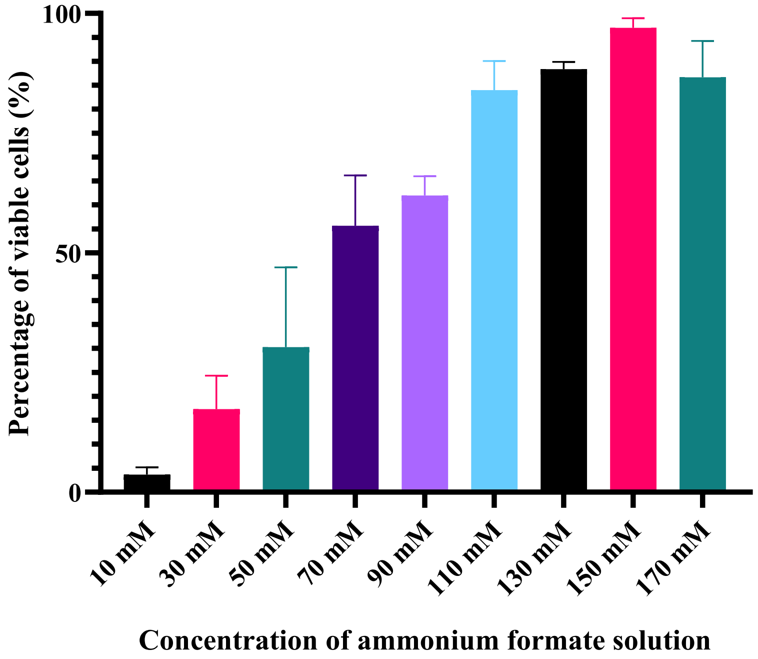

Supplementary Figure S5. Clustered heatmap of single-cell and blank-sample lipidomics data (log transformed and auto scaled) for sampling method comparison. N=10 for capillary sampling single cells and microfluidics single cells. N=3 for mobile phase, microfluidics, and capillary sampling blanks.

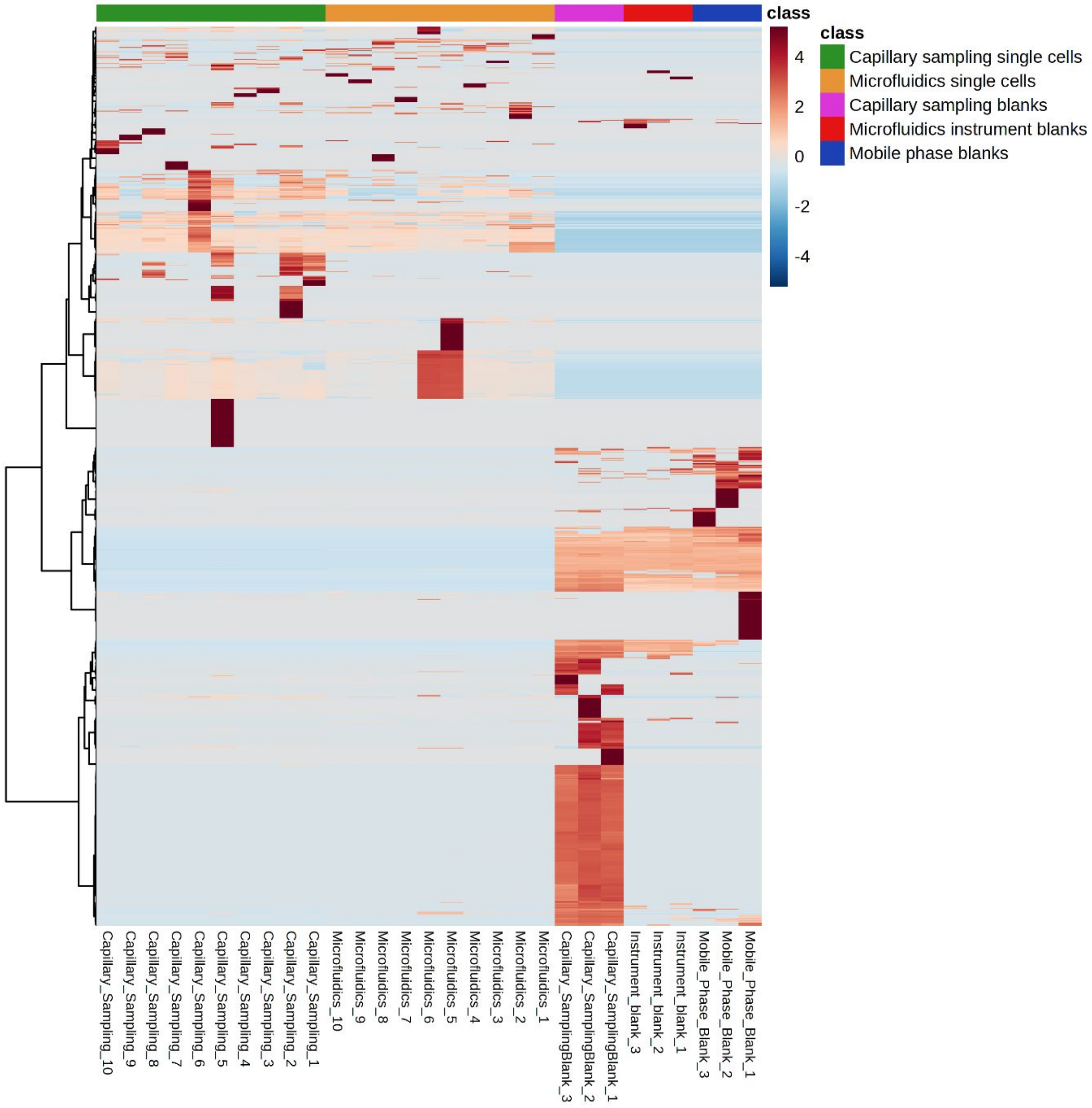

Supplementary Figure S6. Heatmap of blank-sample lipidomics data (log transformed and auto scaled), by lipid class. N=3 for mobile phase, microfluidics, and capillary sampling blanks.

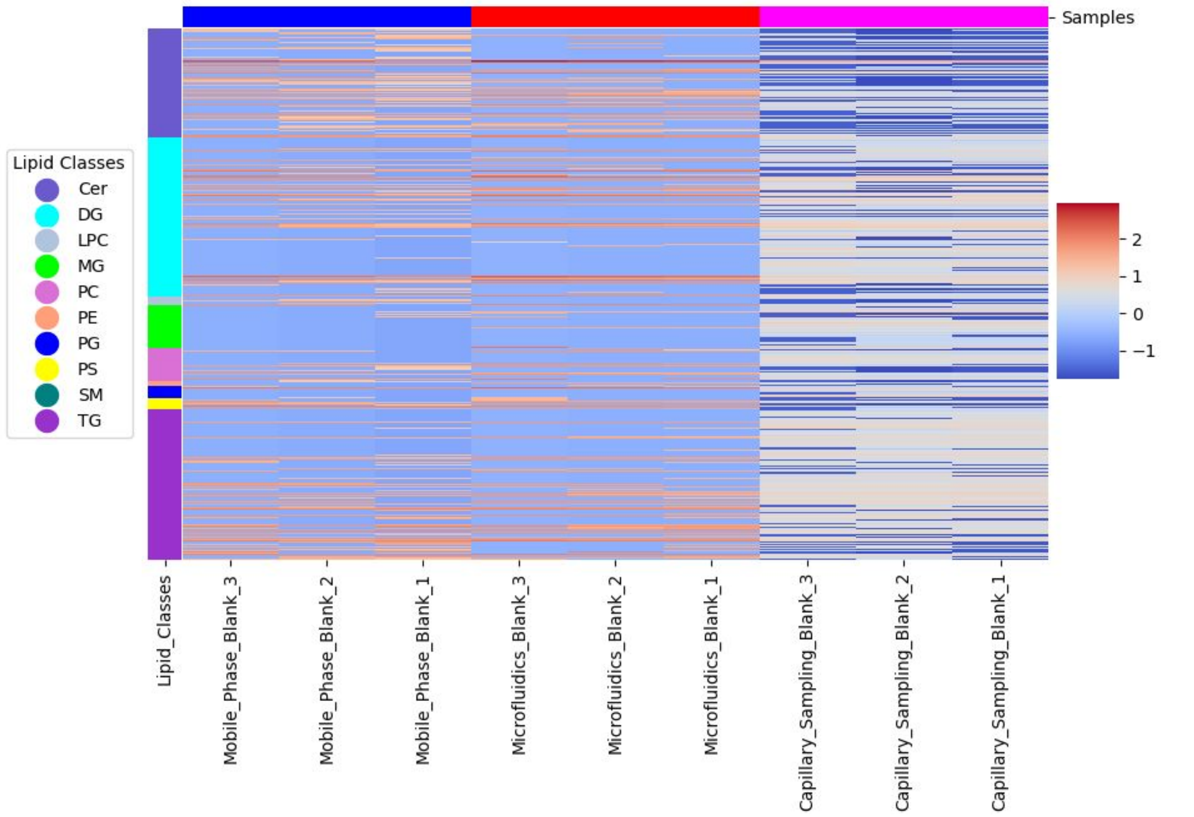

Supplementary Figure S7. Heatmap of single-cell sample lipidomics data (log transformed and auto scaled), by lipid class. N=10 for microfluidics and capillary sampling single cell samples.

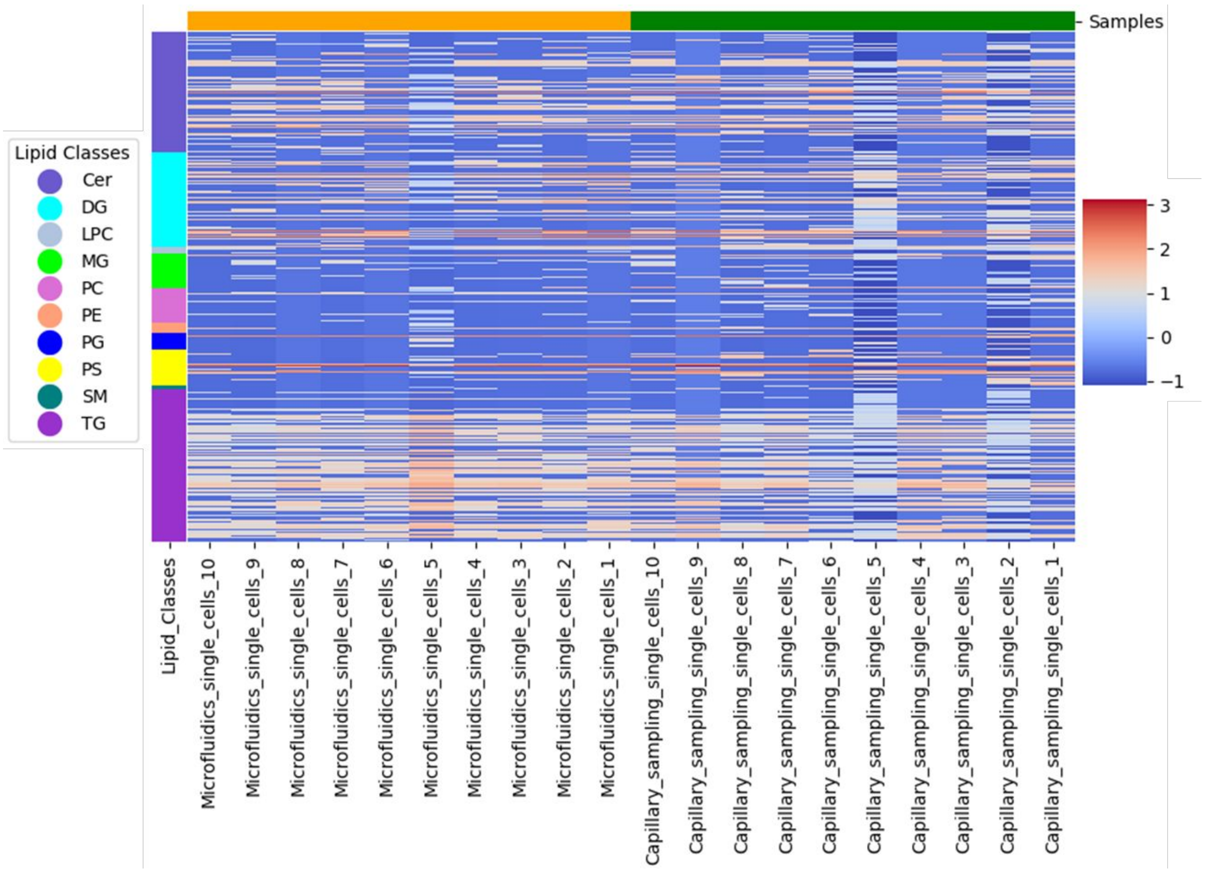

Table S2: Relative standard deviations for internal standard (EquiSPLASH, Avanti UK) lipid classes present in single-cell samples from microfluidics or capillary sampling methods.

	**Capillary Sampling (%)**	**Microfluidics Sampling (%)**
**PC** (15:0-18:1(d7))	7.1	5.0
**LPC** (18:1(d7))	7.1	6.9
**PE** (15:0-18:1(d7))	5.1	4.8
**SM** (d18:1-18:1(d9))	5.7	5.8
C15 **Ceramide** (d7)	8.6	2.9
**PG** (15:0-18:1(d7))	11.0	8.3
**TAG** (15:0-18:1(d7)-15:0)	11.0	7.8
**DAG** (15:0-18:1(d7))	5.4	5.5
**PS** (15:0-18:1(d7))	6.9	5.5
**LPE** (18:1(d7))	13.0	9.6
**PI** 15:0-18:1(d7)	14.0	8.9
**Chol Ester** 18:1(d7)	32	14
**MAG** 18:1(d7)	7.7	7.8
